# How does item wording affect participants’ responses in Likert scale? Evidence from IRT analysis

**DOI:** 10.3389/fpsyg.2024.1304870

**Published:** 2024-10-04

**Authors:** Biao Zeng, Minjeong Jeon, Hongbo Wen

**Affiliations:** ^1^Collaborative Innovation Center of Assessment toward Basic Education Quality, Beijing Normal University, Beijing, China; ^2^Department of Education, School of Education and Information Studies, University of California, Los Angeles, CA, United States

**Keywords:** Likert scale, item wording, method effect, positively worded, negatively worded, bi-factor model, graded response model, IRT

## Abstract

Researchers often combine both positively and negatively worded items when constructing Likert scales. This combination, however, may introduce method effects due to the variances in item wording. Although previous studies have tried to quantify these effects by using factor analysis on scales with different content, the impact of varied item wording on participants’ choices among specific options remains unexplored. To address this gap, we utilized four versions of the Undergraduate Learning Burnout (ULB) scale, each characterized by a unique valence of item wording. After collecting responses from 1,131 college students, we employed unidimensional, multidimensional, and bi-factor Graded Response Models for analysis. The results suggested that the ULB scale supports a unidimensional structure for the learning burnout trait. However, the inclusion of different valences of wording within items introduced additional method factors, explaining a considerable degree of variance. Notably, positively worded items demonstrated greater discriminative power and more effectively counteracted the biased outcomes associated with negatively worded items, especially between the “Strongly Disagree” and “Disagree” options. While there were no substantial differences in the overall learning burnout traits among respondents of different scale versions, slight variations were noted in their distributions. The integration of both positive and negative wordings reduced the reliability of the learning burnout trait measurement. Consequently, it is recommended to use exclusively positively worded items and avoid a mix in item wording during scale construction. If a combination is essential, the bi-factor IRT model might help segregate the method effects resulting from the wording valence.

## 1 Introduction

The Likert scale is one of the most widely used scales in the fields of education and psychology. By presenting a description of a specific event or content, respondents can choose an option that best reflects their sentiment from a range of options, representing varying degrees of agreement, thereby achieving the measurement of the target trait (Likert, 1932). However, extensive research has found that many respondents often display response bias (such as acquiescence bias) when answering Likert scales. As a result, their responses are often shaped by irrelevant factors rather than the target trait, significantly interfering with the reliability of the research results (Cronbach, 1946; Paulhus, 1991).

To address this issue, researchers proposed the combined use of positively and negatively worded items. With varying item wordings, the aim was to create subtle cognitive jolts for the participants, thus prompting them to respond to the scale more diligently (Podsakoff et al., 2003). However, this approach led to new challenges. Different item wordings might bring about additional method factors (Bolin and Dodder, 1990; Wang et al., 2001), and the influence of items with the same wording direction may exhibit strong stability (e.g., cross-country stability) (Boduszek et al., 2021). The understanding and cognitive decision-making processes elicited by positively and negatively worded items may not be consistent, potentially causing a substantive shift in the factor structure of the scale. This poses a significant threat to the construct validity of the scale (Bulut and Bulut, 2022; Tang et al., 2024). Moreover, it considerably diminishes the internal consistency of the scale, thereby severely weakening the reliability of the measurement results (Wang et al., 2015; Zeng et al., 2020).

To better assess the impact brought by different item wordings, researchers have begun to utilize the Factor Analysis framework via two distinct approaches: Exploratory Factor Analysis (EFA) and Confirmatory Factor Analysis (CFA). The EFA method is primarily used to evaluate the number of dimensions in a scale containing both positively and negatively worded items, investigating whether different wordings result in changes in the number and structure of factors. It also further explores the direction and composition of method effects through factor loadings (Zeng et al., 2020; Dodeen, 2023). Conversely, the CFA method is typically employed when existing theories or research have already specified the possible dimensions of the scale (Lindwall et al., 2012; DiStefano and Motl, 2009; Tang et al., 2024). It helps determine whether the structure of the scale conforms to these existing theories or research, providing an empirical method to evaluate the specific performance of hypothesized dimensional structures (Boduszek et al., 2022). For example, Willmott et al. (2018) validated the dimensional framework of the Juror Decision Scale using a confirmatory bi-factor model. In this context, fit indices are used to assess how well the model fits the data, and factor loadings are examined to determine the strength and significance of potential method effects.

Within the CFA framework, the multitrait-multimethod (MTMM) approach is widely used (Campbell and Fiske, 1959). By distinguishing the target trait from the method effects caused by item wording and presuming two distinct factors, it could adequately evaluate the influence of different item wordings through factor loadings. Although numerous studies have affirmed the significant method effects resulting from varying item wordings, there remains no clear answer as to whether positively or negatively worded items induce stronger method effects. Some studies found that negatively worded items yield stronger method effects (Marsh, 1996; Quilty et al., 2006; DiStefano and Motl, 2009; Tang et al., 2024), while many other studies suggested that positively worded items introduce more pronounced method effects (Farh and Cheng, 1997; Lindwall et al., 2012). The emergence of this phenomenon might be attributed to the fact that comparisons of different item wordings are based on diverse scales. Varying test contents might introduce additional measurement errors to the results. Analysis based on the same test content but with different item wording might better address this issue.

More critically, existing studies primarily rely on traditional factor analysis frameworks and solely explore the method effects on the item level caused by item wording through factor loadings (DiStefano and Motl, 2009; Zeng et al., 2020; Bulut and Bulut, 2022; Dodeen, 2023; Tang et al., 2024). For instance, Zeng et al. (2020) utilized the CFA methodology to examine the magnitude of method effects caused by different item wordings at the item level through factor loadings. However, how different valences of item wording influence the specific option choices of participants remains an open question. Answering this question holds significant theoretical and practical implications for test item design and deepens our understanding of how item wording affects the response process. Yet, the existing confirmatory factor models might not adequately address this issue, whereas polytomous Item Response Theory (IRT) models can offer potential solutions. These models provide the step difficulty between each option for every item (Samejima, 1969), allowing for an assessment of how different item wordings might influence participants’ choices of specific options. Furthermore, the existing exploratory and confirmatory factor analysis models are based on limited-information estimation methods, which may limit their estimation accuracy. In contrast, IRT models, utilizing full-information estimation methods, can provide more accurate estimation results (Gibbons et al., 2007).

Currently, researchers are beginning to explore the estimation of method effects caused by positively and negatively worded items through IRT models (Sliter and Zickar, 2014; Wang et al., 2015; Cole et al., 2019). Similar to factor analysis, researchers can classify IRT models into exploratory and confirmatory approaches. In the realm of exploratory IRT approach, this approach primarily utilizes the Exploratory Multidimensional Item Response Theory model to investigate the number and nature of dimensions underlying a scale. Moreover, the effectiveness of this approach in exploring factor structures has been empirically demonstrated (Fontanella et al., 2019).

In the field of confirmatory IRT approach, this method is mainly used for validating the theoretical dimensions or conceptual frameworks of scales and can be used to examine wording effects and other methodological factors. Wang et al. (2015) utilized bi-factor IRT model and found that the wording effect in the Program for International Student Assessment and the Trends in International Mathematics and Science Study was substantial. Ignoring the wording effect resulted in overestimated test reliability and biased person measures. Furthermore, researchers discovered that negatively worded items produced comparatively higher difficulty and lower discrimination parameters than positively worded items and yielded almost no information. The model fit was improved in four out of five scales by removing negatively worded items (Sliter and Zickar, 2014). Based on this, some studies have used three polytomous IRT models to analyze the Perceived Stress Scale and found that using all positively worded items was statistically more favorable (Cole et al., 2019).

Given that the dimensional structure of most scales is already determined in accordance with theoretical constructs or empirical findings, researchers tend to use confirmatory rather than exploratory IRT models to analyze method effects. However, overall, there have been only a few studies using IRT models to investigate method effects caused by the valence of wording, and the results have been somewhat inconsistent. This inconsistency may result from these studies being based on scales with different content, or on positively and negatively worded items within the same scale, while overlooking the significant interference caused by different dimensions and item construct. Additionally, many existing studies using IRT models are predominantly on the basis of a single polytomous IRT model for analysis, but few of them conducted comparisons between different models (such as bi-factor, unidimensional, and multidimensional models). Therefore, the accuracy of these results needs to be further evaluated (Wang et al., 2015).

Building on the foundation and above-mentioned limitations of existing factor analysis and IRT research, this study intends to use scales related to learning burnout adapted from the same test but with different valences of item wording. Given that the potential dimensions of the learning burnout scale have been established (Maslach and Jackson, 1981; Zeng et al., 2020), employing the framework of confirmatory IRT appears to be more appropriate in this content. Therefore, we will utilize the confirmatory Graded Response Model (GRM) for estimation (including bi-factor, unidimensional, and multidimensional models). Our core objectives are to investigate: (a) the structure of learning burnout traits, (b) the impact of item wording on item discrimination and step difficulty parameters, (c) the effects of item wording on latent target traits, and (d) the influence of item wording on the reliability of scales.

By addressing these objectives, we aim to provide a comprehensive understanding into the consequences of varied item wordings, shedding light on how specific wording valences shape participants’ choices among specific options and impact the reliability and validity of the scales. This insight will clarify the specific effects of item wording on participants’ choices between response options, as well as its influence on the overall performance of the scale. Ultimately, these findings will steer more informed scale development and harness the Graded Response Model to effectively segregate potential method effects.

## 2 Method

### 2.1 Participants and instruments

A total of 1,131 college students participated in answering scales, which is consistent with the data in Zeng et al. (2020). Among them, 368 (32.5%) were male and 764 (67.5%) were female; 414 (36.6%) freshmen, 243 (21.4%) sophomores, 66 (6%) juniors, 170 (15.0%) seniors, and 239 (21.1%) graduate students; regarding monthly living expenses, 95 (8.6%) reported “less than 1,000 yuan (RMB),” 517 (47.2%) “1,000–1,500 yuan,” 306 (27.9%) “1,500–2,000 yuan,” and 178 (16.2%) “more than 2,000 yuan,” indirectly reflecting the family economic conditions of these students. The majority of participants were above 18 years old.

We employed the newly revised Undergraduate Learning Burnout (ULB) scale by Zeng et al. (2020), which was adapted from the initial scale developed by Lian et al. (2005). The revised version consists of 20 four-point Likert scale items, with response options as: “1 = strongly disagree,” “2 = somewhat disagree,” “3 = somewhat agree,” and “4 = strongly agree.” The reliability of the revised scale upon its initial application was 0.79, indicating good stability (Zeng et al., 2020).

To investigate the effects of item wording and to eliminate potential errors due to differing target traits and content, we created three additional versions of the ULB scale based on the original revised edition by altering the wording valence of the items. In all versions of the scale, the items with the same number retain the same test content, but the wording may shift between positive and negative phrasing. The original version contained 8 positively worded items (Item 1, 3, 6, 8, 11, 13, 15, 17) and 12 negatively worded items (Item 2, 4, 5, 7, 9, 10, 12, 14, 16, 18, 19, 20). The original-reverse version switched the phrasing of all items from the original version, resulting in 8 negatively worded items (Item 1, 3, 6, 8, 11, 13, 15, 17) and 12 positively worded items (Item 2, 4, 5, 7, 9, 10, 12, 14, 16, 18, 19, 20). The positive version reworded all negatively phrased items, resulting in 20 positively worded items. Conversely, the negative version reformulated all positively phrased items, leading to 20 negatively worded items.

To ensure the semantic equivalence of the four versions of the ULB scale, we invited four experts to review the items with different wording directions based on the original scale and further modified items where the semantics were not consistent. After several rounds of modifications, the experts unanimously agreed that all 20 items with different wording directions have high semantic equivalence. Consequently, we formed the original-reverse, positive, and negative version ULB scales by combining items with different wording valences. To clarify the actual experience of respondents when answering the scales, we randomly selected 10 college students for testing and conducted one-on-one interviews. Through these interviews, students overwhelmingly considered the wording of the questions to be clear and appropriate, with only a few statements raising potential confusion. We made adjustments based on their feedback and finalized the four versions of the ULB scale. Subsequently, we invited 30 graduate students, either pursuing or having obtained master’s or doctoral degrees, to assess the semantic equivalence of 20 positively and negatively worded items. They rated the items on a scale from 1 to 9, where 1 indicate “extremely dissimilar” and 9 indicate “extremely similar.” Higher scores indicate greater semantic similarity. The results revealed that the mean rating for the 20 items was 8.04, with a standard deviation of 0.59, indicating a very high level of semantic equivalence between positively and negatively worded items, with minimal variation in ratings across different items, demonstrating high stability.

For detailed information regarding the design and specific items of the different versions of the scale, please refer to Section 1 in the attached Supplementary materials, as outlined in Supplementary Tables S1, S2.

### 2.2 Data collection

Data was collected using an online survey platform, Sojump. Each participant were free to select any version of the scale they desired. Different versions were merely distinguished by unique letters, ensuring that participants’ choices were completely random.

### 2.3 Analysis

Initially, we cleaned and organized the data. We removed data from 35 participants who exhibited extreme response patterns characterized by highly consistent straightlining behavior following the approach of Arias et al. (2020) and Liu (2019). Subsequently, we retained the response data of 1,096 participants for the final analysis. This consisted of 306 participants in the original version, 258 in the original-reverse version, 277 in the positive version, and 255 in the negative version. Before conducting the analysis, we converted the scoring for negatively worded items across all versions. Therefore, all final data adhered to the same positively worded scoring system. This implies that higher scores indicate students possessing a more positive attitude toward learning and experiencing a lower level of learning burnout. Next, we employed chi-square tests to compare the gender, grade and monthly living expense composition of the four versions of the questionnaire. The results revealed that except for the original version, where the proportion of males and graduate students was slightly higher than in other versions, there were no significant differences among the other three versions. In terms of monthly living expenses, there were no significant differences among participants of all four versions, suggesting that the family economic conditions of these students might be similar. Overall, the participant characteristics across the different versions of the questionnaire are relatively similar.

**Figure 1:**
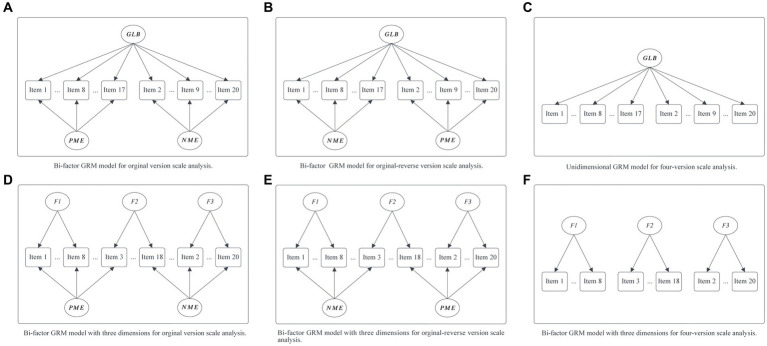
Six sub-models for different versions of the ULB scale under the unidimensional [Model 1: **(A–C)**] and three dimensions [Model 2: **(D–F)**] hypothesis of learning burnout traits. GLB, general learning burnout factor, F1, F2, F3 represent hypothesis three dimensions of ULB scale; PME, method effects produced by positively worded items; NME, method effects produced by negatively worded items (the same as below).

Continuing, some previous studies suggested that the ULB scale possesses a three-dimensional structure (Maslach and Jackson, 1981), while others proposed that it may comprise only a single general learning burnout factor (Zeng et al., 2020). To comprehensively examine whether the ULB scale exhibits a three-dimensional or unidimensional structure, we employed confirmatory three-factor and one-factor GRM models to analyze all four versions of the ULB scale. In particular, to better assess whether combining positively and negatively worded items in the scale introduces significant method effects (factors), it might be more appropriate to use bi-factor models (Willmott et al., 2018), with one or three primary factors representing learning burnout and two secondary factors representing method effects (Gibbons et al., 2007). Therefore, in the analysis of the original and original-reverse versions of the ULB scale, we additionally utilized confirmatory bi-factor models to ensure the robustness of the results and facilitate the subsequent comparison to determine which model is more suitable. Specifically, each of the four versions of the scale had its distinct sub-model, as illustrated in Figure 1. Under the assumption of a one-dimensional learning burnout trait (Model 1), the original version was analyzed by models A and C, the original-reverse version utilized models B and C, and the positive and negative versions were analyzed with model C. Conversely, under the three-dimensional learning burnout trait hypothesis (Model 2), we constructed confirmatory bi-factor and three-dimensional GRM models for the four ULB scale versions. In this scenario, the original version was analyzed by models D and F, the original-reverse version employed models E and F, and the positive and negative versions were analyzed with model F. We utilized the *mirt 1.38.1* package in R and applied the EM algorithm (please refer to the manual) to analyze these models (Chalmers, 2012).

To clarify the effectiveness and robustness of the GRM model for analyzing samples from four versions of the scale (255–306 participants), we simulated response data for 20 items with 250 participants, aligning with our study design. We analyzed the performance of both bi-factor and unidimensional GRM models, conducting 100 replications to obtain robust estimates. The findings indicated that, for both unidimensional and bi-factor GRM models, the overall correlation between the estimated and true parameters for both items and persons exceeds 0.85. This surpasses the recommended threshold for GRM sample size analysis (*N* = 500) by Reise and Yu (1990). Notably, the unidimensional GRM model whose a correlation exceeded 0.92, even surpasses correlation levels found in existing GRM studies with an *N* = 1,000. Additionally, Bias and RMSE values for item and person parameters are minimal, falling below the RMSE recommended for a sample size of 500 by Reise and Yu (1990). It is noteworthy that the RMSE for item difficulty parameters and person latent trait parameters in the unidimensional GRM model approaches the precision observed in studies with a sample size of 1,000 (Reise and Yu, 1990) (see Supplementary Tables S3, S4 for details on the simulation study setup, process, and results). In conclusion, it is rational to use the *mirt* package with the EM algorithm to analyze data containing 20 items and 255–306 participants in this study.

After obtaining the estimation results for each model, we determined the best-fitting model by comparing the Akaike Information Criterion (AIC) and Bayesian Information Criterion (BIC) values. A smaller AIC and BIC values indicate a better fit of the model to the data. We then employed the best fitting model to further analyze the Explained Common Variance (ECV) in the bi-factor model to assess the unidimensionality of the scale. Meanwhile, low ECV values (e.g., < 0.70) indicate the presence of a substantial amount of multidimensionality in the data, implying that when the ECV is lower than 0.7, the method factors brought about by positively and negatively worded items explain a lot of variances, and in this case, the scale is multidimensional. Then, we individually analyze and compare the four scales in terms of item discrimination ( a ), item step difficulty ( b ), participants’ latent learning burnout traits, and method effects. Specifically, item discrimination and difficulty parameters were primarily evaluated to determine the impact of different item wordings on participants’ response tendencies. The discrimination parameter represents its ability to distinguish between participants with varying latent traits in terms of their likelihood of agreeing with an item. The difficulty represents the probability of participants tending to agree with this option, with a lower difficulty meaning that participants of the same ability are more likely to agree with that option.

Next, to better understand the latent learning burnout traits obtained from different versions of the scale, we presented the distribution of latent learning burnout among participants for the four versions of the scale using boxplots and histograms. On this basis, we employed ANOVA to analyze whether there were statistically significant differences between the different versions.

Lastly, we analyzed the reliability of the latent traits across the four versions of the scale using the empirical_rxx function in the mirt1.38.1 package. This analysis was based on the estimated scores and their standard errors (Chalmers, 2012). The purpose of this analysis was to further investigate the impact of different combinations of item wordings on the reliability of the scales.

## 3 Results

### 3.1 Fit indices and explained common variance

We estimated both the unidimensional (Model 1) and three-dimensional (Model 2) latent traits hypothesis models of learning burnout for the four versions of the scale, as shown in Table 1. The results revealed that for both the original and original-reverse version scales, Model 1 had smaller AIC and BIC values. For the negative version scale, Model 1 also had the smallest BIC value. This suggests that Model 1 generally fits better than Model 2, indicating that the learning burnout trait is more likely to be a unidimensional structure. Therefore, we will continue with the assumption of Model 1 for subsequent analyses.

**Table 1:** Fit indices of bi-factor and unidimensional (three-dimensional) GRM models for four versions of the ULB scale.

		Original		Original-reverse		Positive	Negative
		Bi-factor	One/three factor	Bi-factor	One/three factor	One/three factor	One/three factor
AIC	Model 1	12124.52	12680.11	9805.64	10146.57	8981.55	9720.62
	Model 2	12128.23	12720.79	9807.68	10196.79	8892.98	9674.14
BIC	Model 1	12496.88	12977.99	10160.94	10430.8	9271.47	10000.38
	Model 2	12500.59	13018.68	10162.98	10481.02	9255.38	10024.72

The ULB scale across the four versions is based on different sub-scale models within both Model 1 and Model 2, as detailed in the “Method” section.

We found that in Model 1, the best fitting models for the original and original-reverse versions were the bi-factor models, which included one unidimensional learning burnout factor and two method effect factors related to positively and negatively worded items separately, while the positive and negative versions were modeled as one-factor models, which only included one unidimensional learning burnout factor. Then, we analyzed the ECV of the bi-factor model for the original and original-reverse version scales. The results revealed that the ECVs for the two scales were 0.25 and 0.41, respectively, far below the unidimensionality standard of 0.7. This suggests that the factors brought by positively and negatively worded items explained a large amount of variances, which may somewhat affect the factor structure of the two versions of the ULB scale.

### 3.2 Item parameter

The estimated item parameters for the four versions of the ULB scale for these models are presented in Tables 2, 3. Initially, we utilized an independent sample t-test to compare the item parameters between the positively and negatively worded items within the original and original-reverse scales based on different item content. The findings indicated that positively worded items have a higher discriminatory power when assessing students’ learning burnout traits.

**Table 2:** Item parameters of the original and original-reverse ULB scale for the bi-factor Graded Response Model.

	*a*			*b*		
	GLB	PME	NME	1	2	3
Original version						
Item 1	−0.35	1.83		−1.86	0.37	2.09
Item 2	0.00		1.06	−0.32	2.08	3.44
Item 3	0.46	1.69		−2.25	0.20	2.11
Item 4	−0.23		1.92	−1.25	0.36	1.80
Item 5	−0.53		2.30	−1.59	−0.07	1.75
Item 6	0.55	1.15		−2.24	0.53	2.65
Item 7	0.09		1.28	−2.36	−0.24	2.03
Item 8	−0.06	1.57		−2.23	−0.17	2.06
Item 9	−0.14		2.18	−1.14	0.78	2.36
Item 10	−0.88		1.24	−1.53	0.29	2.46
Item 11	0.32	1.64		−1.91	0.70	2.65
Item 12	−0.44		1.11	−1.39	0.65	3.05
Item 13	0.25	1.29		−1.80	0.74	2.44
Item 14	−0.65		1.29	−2.66	−0.88	2.00
Item 15	0.33	1.52		−1.66	0.59	2.57
Item 16	−1.36		1.38	−1.19	0.31	2.16
Item 17	0.16	1.79		−2.34	0.30	2.31
Item 18	−0.93		1.86	−1.76	−0.12	2.08
Item 19	−3.40		2.06	−1.17	0.14	1.72
Item 20	−0.78		1.54	−1.96	−0.19	1.79
Original-reverse version						
Item 1	1.22		1.10	−1.46	0.58	2.58
Item 2	0.68	0.99		−1.22	1.35	3.43
Item 3	0.19		1.79	−1.74	0.54	2.57
Item 4	1.04	1.76		−1.80	0.13	1.84
Item 5	1.84	2.14		−1.63	0.15	2.08
Item 6	−0.14		0.81	−2.95	0.47	4.05
Item 7	0.38	1.49		−3.11	−0.71	1.50
Item 8	−0.39		0.77	−4.05	−0.41	3.16
Item 9	0.85	1.90		−2.12	−0.06	2.28
Item 10	1.73	0.93		−1.75	0.20	2.26
Item 11	0.30		1.84	−1.16	1.12	2.70
Item 12	0.60	0.92		−2.34	0.29	2.69
Item 13	0.17		1.25	−1.23	1.02	2.94
Item 14	1.19	1.87		−1.80	0.44	2.88
Item 15	0.32		1.85	−0.32	1.28	3.04
Item 16	1.48	0.97		−1.89	0.26	2.39
Item 17	0.45		1.34	−2.53	0.44	3.09
Item 18	1.26	1.51		−1.74	0.64	2.73
Item 19	3.49	0.91		−1.35	0.48	2.38
Item 20	−0.01	1.26		−2.98	−0.56	1.73

*a, stands for the discrimination parameter; b, represents the difficulty parameter.*

**Table 3:** Item parameters of the positive and negative ULB scale for the unidimensional Graded Response Model.

	*a*	*b*		
	GLB	1	2	3
Positive version				
Item 1	1.58	−2.51	0.64	3.09
Item 2	1.06	−1.71	1.84	4.52
Item 3	1.61	−2.92	0.03	2.76
Item 4	1.45	−2.76	−0.06	2.61
Item 5	2.43	−2.14	0.01	2.54
Item 6	1.27	−2.06	0.78	3.34
Item 7	1.78	−2.92	−0.67	2.33
Item 8	1.05	−3.57	−0.19	3.32
Item 9	1.51	−3.15	−0.19	2.58
Item 10	1.64	−1.95	−0.03	2.70
Item 11	1.72	−2.24	0.67	2.98
Item 12	1.13	−2.50	0.60	2.95
Item 13	1.52	−1.85	0.90	3.31
Item 14	2.46	−1.91	0.55	2.92
Item 15	1.28	−2.28	0.61	3.70
Item 16	1.75	−2.05	−0.07	2.85
Item 17	2.40	−2.33	0.12	2.26
Item 18	2.32	−2.12	0.26	2.68
Item 19	1.72	−2.34	0.46	2.99
Item 20	1.24	−3.60	−0.23	2.51
Negative version				
Item 1	1.41	−1.33	0.84	3.20
Item 2	0.95	0.06	2.84	
Item 3	1.88	−1.30	0.76	2.48
Item 4	1.74	−1.12	0.59	2.26
Item 5	2.97	−1.34	0.12	1.68
Item 6	1.55	−1.31	0.50	2.40
Item 7	1.40	−2.08	0.07	2.20
Item 8	1.56	−1.60	0.32	2.13
Item 9	2.50	−0.96	0.84	2.25
Item 10	1.68	−1.37	0.34	2.48
Item 11	1.92	−0.85	1.31	2.98
Item 12	−0.06	35.95	2.67	−39.58
Item 13	1.57	−0.72	1.23	3.07
Item 14	2.02	−1.52	−0.26	1.72
Item 15	1.09	−0.59	2.14	4.19
Item 16	1.39	−1.27	0.66	2.95
Item 17	2.49	−1.39	0.39	2.16
Item 18	2.57	−1.23	0.20	1.82
Item 19	2.07	−1.20	0.39	2.22
Item 20	1.72	−1.58	0.15	1.78

No participants chose the “4 (strongly agree)” option on Item 2, so the difficulty of step 3 is null in the table.

Furthermore, we observed that in the original and original-reverse versions of the scale, which combine positively and negatively worded items, many negatively worded items and very few positively worded items showed negative discrimination parameters on the general learning burnout factor. This suggests that negatively worded items may have a more adverse impact on distinguishing between students at different levels of the learning burnout trait. However, in the single valence of wording positive and negative versions, almost no item showed a negative discrimination on the general learning burnout factor. To mitigate potential interference arising from different models (bi-factor and unidimensional GRM) for various versions of the scale, we further employed the unidimensional GRM model to analyze both the original and original-reverse versions of the ULB scale. The detailed item parameter results can be found in Supplementary Table S5. The findings indicated that, even when employing the same unidimensional GRM model, both the original and original-reverse versions—incorporating both positively and negatively worded items–still demonstrated numerous items with negative discrimination parameters on the general learning burnout factor. This suggests that the additional method effect introduced by combining positively and negatively worded items interferes with the measurement of the learning burnout target trait, potentially revealing an opposite pattern in distinguishing students with different levels of learning burnout traits.

Notably, the difficulty parameter $b_{1}$ for positively worded items in the original version was significantly lower than that for negatively worded items ( t=−2.56,p<0.05 ). This implies that, compared to negatively worded items, for positively worded items, the step difficulty between options “1 (strongly disagree)” and “2 (disagree)” is smaller. Hence, respondents with similar abilities are more likely to choose option “2” over option “1,” resulting in higher scores, which in turn signifies a lower level of learning burnout.

Subsequently, we employed an ANOVA to compare item parameters across the four scales based on the same item content but different item wording valences. First, regarding the item discriminations, when items changed from negatively worded to positively worded, there were significant differences in the discrimination of items with different wording directions within the general learning burnout factor (*F* = 32.42, *p* < 0.001). Tukey’s *post hoc* test revealed that both the positively worded items in the original-reverse and positive versions had significantly higher discrimination than the negatively worded items in the original version. This indicated that positively worded items contribute to better discrimination in measuring learning burnout traits.

In terms of the difficulty, when item wording was changed from negatively worded to positively worded, there were significant differences in the difficulty of items with different wording directions within $b_{1}$ (*F* = 7.04, *p* < 0.01). Tukey’s *post hoc* test revealed that the difficulty of positively worded items in the positive version was significantly lower than the difficulty of negatively worded items in the original version ( $M_{\mathrm{diff}}$ = −0.90, *p* < 0.01), indicating a decrease in item difficulty. In this scenario, respondents were more inclined to choose the “2 (disagree)” option between choices “1” and “2,” resulting in a higher test score, which symbolizes a lower level of learning burnout. Conversely, when item wording was changed from positively worded to negatively worded, there were also significant differences in the difficulty of items with different wording directions within $b_{1}$ (*F* = 3.62, *p* < 0.05). Tukey’s *post hoc* test indicated that the difficulty of negatively worded items in the negative version was marginally significantly higher than the difficulty of positively worded items in the original version ( $M_{\mathrm{diff}}$ = 0.90, *p* = 0.056). In this case, respondents were more likely to choose the “1 (strongly disagree)” option between choices “1” and “2,” resulting in a lower test score, which represents a higher level of learning burnout.

In summary, the results suggest that positively worded items possess higher discriminatory power. They appear more capable of avoiding the biases introduced by negatively worded items, which significantly skew results toward a higher level of learning burnout, especially when participants choose between the options “strongly disagree” and “disagree.” This distinction is particularly salient.

### 3.3 Latent traits

**Figure 2:**
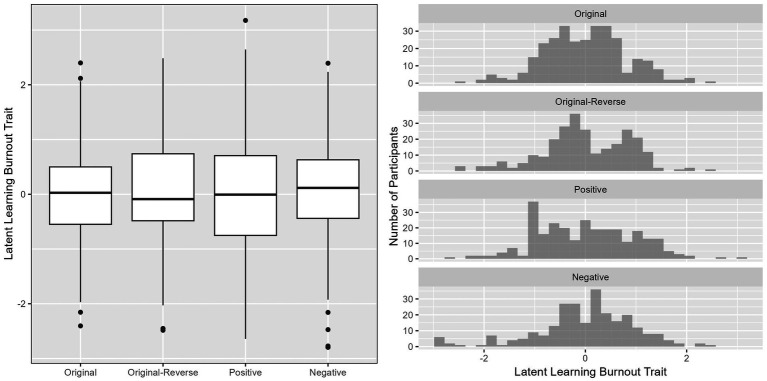
Boxplot and histogram of latent learning burnout traits for participants across four versions of ULB scale.

We used boxplots and histograms, as shown in Figure 2, to visualize the distribution of participants’ latent learning burnout traits across the four versions of the ULB scale. The analysis revealed that, on the whole, students’ latent learning burnout levels were quite similar across all four scale versions. Notably, the positive version of the scale exhibited a higher prevalence of lower learning burnout levels, possibly due to its positively worded item format. Expanding upon these results, we conducted an ANOVA to statistically investigate potential differences in participants’ latent learning burnout traits among the four scale versions. The outcomes of the analysis indicated that there were no significant differences in participants’ latent learning burnout levels across the different scale versions [*F* (3, 1,092) = 0.0001, *p* = 1]. This suggests that the measurement of students’ learning burnout levels remained relatively consistent across the various versions of the ULB scale.

### 3.4 Reliability

Finally, we calculated the reliability of the different latent traits based on participants’ latent traits and their standard errors to explore the impact of different item wording on the reliability of the scales. Results in Table 4 showed that when the ULB scale contained only a single type of item wording (either positively worded or negatively worded), it achieved a high level of reliability. However, when we employed a mix of positively and negatively worded item formulations, this introduced additional method effects. These method effects significantly undermined the reliability estimates of the learning burnout trait, leading to a substantial drop in the scale reliability.

**Table 4:** Reliability of latent trait in four different versions of the ULB scale on the best-fitting model.

	GLB	PME	NME
Original	0.65	0.81	0.82
Original-reverse	0.73	0.74	0.77
Positive	0.92		
Negative	0.93		

## 4 Discussion

This study explored the influence of different item wording methods on participants’ responses to the ULB scale. Compared with the existing study, our utilization of the unidimensional, multidimensional, and bi-factor GRM model allowed for a more nuanced exploration, extending the analysis of method effects from item level to option level. Building on this foundation, we further explored the influence of item wording on participants’ latent target traits and the scale’s reliability.

Firstly, the results from all four versions of the ULB scale predominantly supported the single-factor structure of learning burnout, which is consistent with existing research (Zeng et al., 2020). Moreover, the combination of positively and negatively worded items introduced additional method factors, and these method factors substantially explained the variance in the variables. Based on this foundation, we compared different versions of the scale to investigate the impact of varying item wording methods. The results showed that positively worded items possess higher discrimination than negatively worded items. This means that positively worded items can better differentiate respondents with different levels of learning burnout and have superior measurement capability. This finding bolsters the superior performance of positively worded items as supported by previous studies (Farh and Cheng, 1997; Lindwall et al., 2012; Dodeen, 2023).

Furthermore, we observed that using the original and original-reverse ULB scales, which contain both positively and negatively worded items, resulted in items exhibiting negative discrimination in measuring the target learning burnout traits. Importantly, irrespective of employing either the bi-factor or unidimensional GRM model, numerous items exhibited negative discrimination, a phenomenon absent in positive and negative versions featuring a singular approach to item wording (positively or negatively worded only). This occurrence was likely attributed to the additional method effect factors brought by the combination of different item wording approaches (Marsh et al., 2010; Lindwall et al., 2012; Zeng et al., 2020), which disrupted the scale’s ability to distinguish respondents with different levels of learning burnout, thereby significantly reducing the validity of the measurement results. Moreover, the fact that many negatively worded items showed negative discrimination in the general learning burnout scale suggests that negatively worded items have a greater adverse impact on the measurement properties of the scale (DiStefano and Motl, 2009; Sliter and Zickar, 2014; Dodeen, 2023).

More critically, by comparing items with different contents and item wordings within the same scale, as well as items with the same content but different item wordings across different scales, we obtained similar results. Specifically, positively worded items can better mitigate the bias toward higher levels of learning burnout potentially caused by negatively worded items, especially when respondents choose between “strongly disagree” and “disagree” options. This phenomenon might arise because negatively worded items possibly require a more complex cognitive decision-making process. Moreover, negatively worded items might introduce semantic comprehension difficulties, leading respondents to show higher level of learning burnout (Podsakoff et al., 2003). In contrast, positively worded items can avoid these potential interferences more effectively, providing more accurate estimates of the target trait (Cole et al., 2019; Zeng et al., 2020; Dodeen, 2023; Tang et al., 2024).

Moreover, we delved into the distribution patterns of latent target traits. Respondents from different version scales exhibited relatively consistent overall distributions of latent learning burnout traits, but there were variations in the distributions at different trait levels. For instance, the positive version exhibited a higher prevalence of lower learning burnout levels, which further highlights the characteristics of positively worded items (Cole et al., 2019; Dodeen, 2023; Tang et al., 2024).

Lastly, the reliability of scales mixing positively and negatively worded items significantly decreased, further attesting to the negative impact of additional method factors introduced by different item wordings. This finding aligns closely with existing research (Lee et al., 2008; Zeng et al., 2020). In light of these results, we recommend utilizing solely positively worded items when constructing assessments to ensure superior psychometric properties and to better evade the methodological issues and reliability reduction associated with mixing positively and negatively worded items (Dodeen, 2023). If a mix of positively and negatively worded items is necessary, we advocate adopting the bi-factor framework (Willmott et al., 2018; Tang et al., 2024), particularly bi-factor models based on IRT, for separating method effects from the target trait, thereby achieving a more accurate estimation of the target trait.

### 4.1 Limitations and future directions

While this study provides valuable insights, it also has certain limitations. Firstly, the research primarily focused on the learning burnout domain, and the sample size used was limited. Future studies are expected to sample a larger population and validate the findings of this research in Likert scales from other domains. Secondly, the study employed a common item design, and some versions of scale involved the same anchor items. In the future, it might be beneficial to further utilize linking and equating methods, placing item parameters and participants’ latent abilities on the same scale, which might yield more intriguing and precise results.

Additionally, some studies suggested that variations in the psychometric properties of scales may result from inconsistent respondents. These participants are sometimes attributed to being careless or insufficient effort (C/IE). Although the between-subjects design in this study may not be suitable for applying the Factor Mixture Model (FMM) to analyze respondents with inconsistent patterns, conducting this analysis remains valuable. Data could be collected using a within-subjects design to better analyze potential C/IE participants with the new FMM method in the future, thereby more accurately estimating the impact of different wording valences on items (Arias et al., 2020).

Furthermore, comparing models requires careful consideration, although our study lends greater support to the bi-factor model based on fit indices. The ECV for the general factor of the original and original-reverse versions of the ULB scale was low (0.25 and 0.41), significantly lower than the 80% explanation rate for the general factor in the Rosenberg Self-Esteem Scale reported in an existing study (Reise et al., 2016). This finding potentially supports a multidimensional model and suggests that a portion of participants may be explained by the bi-factor model. However, determining which percentage of participants can be modeled with bi-factor specifications and which percentage can be explained by a unidimensional model is a worthwhile endeavor for future research (Reise et al., 2016).

Finally, our study revealed that combining positively worded and negatively worded items can lead to a negative discrimination in many items. In the future, we can apply the Nominal Response Model to assess the category boundary discrimination of each item (Preston et al., 2011). This approach may provide valuable insights into whether these items are genuinely ordered and how individuals with varying levels of learning burnout select response options in the ULB scale.

## Data Availability

The raw data supporting the conclusions of this article will be made available by the authors, without undue reservation.
