# Supplementary material for: How does item wording affect participants’ responses in Likert scale? Evidence from IRT analysis
Source: Front Psychol. 2024 Oct 4;15:1304870. doi: 10.3389/fpsyg.2024.1304870 (PMC11486723; doi:10.3389/fpsyg.2024.1304870)
Supplement: Supplementary file 1 [file Data_Sheet_1.pdf]

Supplementary Material

1 Supplementary information on the four versions of ULB scale

In the original ULB Scale, there were 8 positively worded items (Block 1, PW) and 12 negatively worded items (Block 2, NW). The original-reverse version of the scale modified the phrasing of all items from the original version, converting positively worded items into negatively worded ones and negatively worded items into positively worded ones. For instance, Item 3, which originally read “mastering professional knowledge is easy for me” was changed to “mastering professional knowledge is difficult for me”, and Item 10, which stated “I rarely study after class” became “I often study after class”. As a result, this version consisted of 8 negatively worded items (Block 1, NW) and 12 positively worded items (Block 2, PW). The positive version of the scale contained 20 positively worded items, while the negative version contained 20 negatively worded items. The design and relationships of these four versions were shown in Supplementary Table S1. The specific wording differences for each item in the ULB scale were presented in Supplementary Table S2.

Supplementary Table S1. The structural design and relationships among the four versions of the ULB Scale.

PW (Block 1)	NW (Block 2)	NW (Block 1)	PW (Block 2)
Original	Original		
		Original-reverse	Original-reverse
Positive			Positive
	Negative	Negative	

Note. PW = Positively worded; NW = Negatively worded; Original = Original version; Original-reverse = Original-reverse version; Positive = Positively worded only version; Negative = Negatively worded only version (the same as below).

Supplementary Table S2. The items in ULB scale with varied valence of wording.

Item	Original version	Original-reverse version
1	I have my own study plan and can put it into practice.	I do not have my own study plan and can't put it into practice.
2	I feel the knowledge I learn is useless.	I feel the knowledge I learn is useful.
3	Mastering professional knowledge is easy for me.	Mastering professional knowledge is difficult for me.
4	Waking up in the morning and thinking about facing a day of study, I feel very tired.	Waking up in the morning and thinking about facing a day of study, I feel very energetic.
5	It is difficult for me to maintain a long-term passion for studying.	It is easy for me to maintain a long-term passion for studying.
6	When studying, I can calmly handle my emotional issues.	When studying, I cannot calmly handle my emotional issues.
7	After a whole day of studying, I feel completely exhausted.	After a whole day of studying, I still feel very energetic.
8	Up to now, university studies have fully demonstrated my abilities.	Up to now, university studies have not fully demonstrated my abilities.
9	I feel bored with studying.	I do not feel bored with studying.
10	I rarely study after class.	I often study after class.
11	I can handle the courses in university.	I cannot handle the courses in university.
12	I often doze off while studying.	I rarely doze off while studying.
13	I am very interested in my major.	I am not interested in my major at all.
14	I do not have enough patience in studying.	I have enough patience in studying.
15	It is easy for me to obtain a degree.	It is difficult for me to obtain a degree.
16	I only study when exams are approaching.	I often study even when there are no exams.
17	I am full of energy when I study.	I feel very tired when I study.
18	I want to study but find it boring.	I want to study and find it interesting.
19	I seldom plan and arrange my study time.	I often plan and arrange my study time.
20	Exams always make me feel annoyed.	Exams never make me feel annoyed.

2 Simulation study on the GRM Analysis

In this simulation study, we utilized the *simdata* function from the *mirt 1.38.1* package to generate response data based on both the bi-factor and unidimensional GRM models. Following the specifications outlined by Reise & Yu (1990), we set the slope parameter (a) to follow a uniform distribution between (-2, 2), and the intercept parameters (d) to follow a uniform distribution between (-3, 3). Additionally, participant latent traits (θ) were set to follow a standard normal distribution with a mean of 0 and a standard deviation of 1.

It is important to explain that the *simdata* function consistently uses the intercept (d) parameter to generate the bi-factor data. To ensure accurate comparisons between the estimated values and the true values, and to obtain precise Bias and RMSE results, we reported the simulation study results using the d parameter. In the GRM model, the intercept parameter (d) can be mathematically converted into the difficulty parameter (b), as both parameters change in a synchronized manner. In other words, if the model accurately estimates the d parameter in the GRM, it means that the estimation of the b parameter is also accurate.

Subsequently, using the *mirt 1.38.1* package and the EM algorithm, we conducted separate analyses based on the bi-factor and unidimensional GRM models with 100 replications. Evaluation metrics for assessing simulation estimation performance included Bias, RMSE, and the correlation between estimated parameters and true parameters. The specific results were shown in Supplementary Tables S3 and S4.

Supplementary Table S3. Average Bias and RMSE for data simulated from unidimensional GRM model.

	Bias				RMSE			
	a	*d*			*a*	*d*		
	GLB	1	2	3	GLB	1	2	3
Item 1	0.02	0.03	0.00	-0.04	0.14	0.19	0.15	0.19
Item 2	0.01	0.04	0.01	-0.04	0.16	0.19	0.13	0.20
Item 3	0.04	0.06	0.02	-0.03	0.15	0.20	0.14	0.16
Item 4	0.03	0.04	0.01	-0.03	0.14	0.18	0.14	0.18
Item 5	0.02	0.00	0.00	-0.04	0.14	0.18	0.13	0.19
Item 6	0.04	0.06	0.00	-0.04	0.17	0.18	0.14	0.19
Item 7	0.05	0.10	0.03	-0.03	0.16	0.21	0.15	0.16
Item 8	0.00	0.07	0.03	0.02	0.13	0.17	0.14	0.16
Item 9	0.03	0.02	0.03	-0.01	0.15	0.20	0.14	0.18
Item 10	0.03	0.02	0.01	-0.01	0.14	0.19	0.14	0.19
Item 11	0.02	0.05	0.04	-0.01	0.13	0.21	0.14	0.19
Item 12	0.02	0.07	-0.01	-0.04	0.14	0.21	0.13	0.14
Item 13	-0.02	0.03	-0.01	-0.01	0.15	0.20	0.13	0.18
Item 14	0.03	0.08	0.03	-0.03	0.16	0.19	0.13	0.16
Item 15	0.00	-0.02	-0.01	0.00	0.15	0.20	0.14	0.17
Item 16	0.04	0.07	0.02	-0.03	0.16	0.18	0.12	0.17
Item 17	-0.02	-0.02	-0.01	-0.01	0.14	0.17	0.14	0.17
Item 18	0.02	0.02	0.02	-0.04	0.15	0.19	0.12	0.17
Item 19	0.03	0.07	0.03	-0.04	0.16	0.18	0.14	0.19
Item 20	0.02	0.05	0.01	-0.01	0.16	0.20	0.13	0.18
Average	0.02	0.04	0.01	-0.02	0.15	0.19	0.14	0.18

Note. We conducted 100 replications, and the Bias and RMSE presented here are the average results across these replications.

Supplementary Table S4. Average Bias and RMSE for data simulated from bi-factor GRM model.

	Bias						RMSE					
	a			*d*			*a*			*d*		
	GLB	PME	NME	1	2	3	GLB	PME	NME	1	2	3
Item 1	0.00	0.03		-0.01	0.01	-0.05	0.21	0.20		0.22	0.18	0.22
Item 2	0.04	0.12		0.09	-0.02	-0.07	0.23	0.26		0.23	0.18	0.23
Item 3	0.03	0.08		0.07	0.01	-0.07	0.20	0.24		0.21	0.17	0.23
Item 4	0.04	0.01		0.08	0.02	0.01	0.21	0.21		0.22	0.18	0.22
Item 5	0.02	0.01		0.03	0.02	0.00	0.22	0.22		0.21	0.16	0.20
Item 6	-0.01	0.04		0.07	0.01	-0.03	0.19	0.21		0.23	0.15	0.26
Item 7	0.03	0.07		0.10	0.02	-0.07	0.22	0.23		0.24	0.16	0.18
Item 8	0.02	0.07		0.07	0.01	-0.06	0.21	0.25		0.24	0.18	0.21
Item 9	0.07		-0.04	0.06	0.00	-0.04	0.22		0.21	0.22	0.15	0.23
Item 10	0.09		-0.01	0.07	0.03	-0.02	0.22		0.24	0.18	0.18	0.20
Item 11	0.03		-0.04	0.03	-0.01	-0.01	0.19		0.29	0.20	0.17	0.21
Item 12	0.07		-0.01	0.06	0.03	-0.04	0.23		0.22	0.23	0.15	0.20
Item 13	0.04		-0.03	0.02	0.00	0.00	0.23		0.22	0.22	0.16	0.18
Item 14	0.05		-0.02	0.09	0.02	-0.03	0.20		0.24	0.23	0.15	0.17
Item 15	0.06		0.02	0.09	0.00	-0.05	0.25		0.26	0.22	0.16	0.19
Item 16	0.05		-0.03	0.04	0.01	-0.05	0.23		0.22	0.20	0.14	0.20
Item 17	0.01		0.04	0.05	0.00	-0.07	0.21		0.24	0.22	0.14	0.23
Item 18	0.04		-0.01	0.06	-0.01	-0.05	0.22		0.20	0.20	0.14	0.22
Item 19	0.05		-0.08	0.03	0.01	0.02	0.20		0.25	0.22	0.17	0.22
Item 20	0.00		-0.01	0.03	0.02	0.00	0.21		0.22	0.18	0.15	0.20
Average	0.04	0.06	-0.02	0.06	0.01	-0.03	0.22	0.23	0.23	0.22	0.16	0.21

3 Additional supplementary tables

Supplementary Table S5. Item parameters of the original and original-reverse ULB scale for the one factor GRM model.

	a	*b*		
	GLB	1	2	3
		Original version		
Item 1	0.24	-9.87	1.96	11.07
Item 2	0.92	-0.36	2.30	3.84
Item 3	-0.19	15.38	-1.38	-13.84
Item 4	1.73	-1.31	0.37	1.89
Item 5	2.21	-1.62	-0.07	1.79
Item 6	-0.17	13.67	-3.18	-15.85
Item 7	1.06	-2.71	-0.27	2.30
Item 8	-0.20	13.13	1.03	-11.68
Item 9	1.86	-1.21	0.84	2.52
Item 10	1.47	-1.55	0.30	2.52
Item 11	-0.16	14.50	-5.30	-19.81
Item 12	1.23	-1.36	0.63	2.98
Item 13	0.08	-21.98	9.30	30.21
Item 14	1.49	-2.61	-0.86	1.96
Item 15	-0.20	9.85	-3.55	-15.09
Item 16	1.72	-1.27	0.31	2.27
Item 17	0.19	-16.33	2.12	15.63
Item 18	2.16	-1.72	-0.12	2.05
Item 19	1.91	-1.46	0.15	2.14
Item 20	1.77	-1.92	-0.19	1.76

Supplementary Table S5 (continued). Item parameters of the original and original-reverse ULB scale for the one factor GRM model.

	a	*b*		
	GLB	1	2	3
		Original-reverse version		
Item 1	0.64	-2.82	1.08	5.33
Item 2	1.23	-1.20	1.34	3.36
Item 3	0.26	-8.39	2.65	12.33
Item 4	1.95	-1.85	0.14	1.89
Item 5	2.86	-1.65	0.16	2.07
Item 6	-0.05	48.14	-7.40	-65.10
Item 7	1.17	-3.68	-0.82	1.78
Item 8	-0.26	12.39	1.21	-9.36
Item 9	1.82	-2.26	-0.05	2.42
Item 10	1.71	-1.86	0.19	2.42
Item 11	0.31	-4.67	4.58	11.28
Item 12	1.07	-2.38	0.30	2.74
Item 13	0.25	-4.82	3.92	11.69
Item 14	2.20	-1.83	0.45	2.86
Item 15	0.21	-1.65	7.93	19.15
Item 16	1.63	-1.97	0.26	2.50
Item 17	0.93	-3.43	0.60	4.12
Item 18	1.98	-1.75	0.64	2.71
Item 19	1.81	-1.66	0.55	3.12
Item 20	0.84	-4.03	-0.69	2.35
